# Genetic Complexity of Fusidic Acid-Resistant Small Colony Variants (SCV) in *Staphylococcus aureus*

**DOI:** 10.1371/journal.pone.0028366

**Published:** 2011-11-29

**Authors:** Jonas Lannergård, Sha Cao, Tobias Norström, Alejandro Delgado, John E. Gustafson, Diarmaid Hughes

**Affiliations:** 1 Uppsala University, Department of Medical Biochemistry and Microbiology, The Biomedical Center, Uppsala, Sweden; 2 Uppsala University, Department of Cell and Molecular Biology, The Biomedical Center, Uppsala, Sweden; 3 Microbiology Group, Department of Biology, New Mexico State University, Las Cruces, New Mexico, United States of America; University of Edinburgh, United Kingdom

## Abstract

FusE mutants are fusidic acid-resistant small colony variants (SCVs) of *Staphylococcus aureus* that can be selected with aminoglycosides. All FusE SCVs have mutations in *rplF*, encoding ribosomal protein L6. However, individual FusE mutants including some with the same mutation in *rplF* display auxotrophy for either hemin or menadione, suggesting that additional mutations are involved. Here we show that FusE SCVs can be divided into three genetic sub-groups and that some carry an additional mutation, in one of the genes required for hemin biosynthesis, or in one of the genes required for menadione biosynthesis. Reversion analysis and genome sequencing support the hypothesis that these combinations of mutations in the *rplF*, *hem*, and/or *men* genes can account for the SCV and auxotrophic phenotypes of FusE mutants.

## Introduction

The small colony variant (SCV) phenotype of *Staphylococcus aureus*, is associated with reduced susceptibility to aminoglycosides and auxotrophy for hemin, menadione, thiamine or thymidine [1]. *S. aureus* SCVs are often isolated from patients with persistent recurring infections such as chronic osteomyelitis and skin and soft-tissue infections. These pathogenic traits have been attributed to the ability of SCVs to invade and survive inside nonprofessional phagocytes, such as epithelial and endothelial cells, where they are thereby shielded from the immune response. [1]

The phenotypes associated with *S. aureus* SCV's are thought to result from a dysfunctional electron transport system [1], [2], [3], [4], [5], [6]. These phenotypes include reduced susceptibility to aminoglycoside antibiotics due to alterations in the proton motive force on which they depend for drug uptake, and selection with aminoglycosides can be used to isolate SCV mutants [7]. Because of their very slow growth rates in the laboratory, SCVs are susceptible to overgrowth by faster-growing variants arising from within these mutant populations. The observation of phenotypic instability among many SCV strains initially led to controversy over whether SCV's were phenotypic variants or genetic mutants, however it is now clear that certain genetic mutations lead to the SCV phenotype. Thus, selected or constructed mutations in the following genes have been shown to lead to the SCV phenotype: delta-aminolevulinic acid dehydratase (*hemB*) [8], [9], ferrochetalase (*hemH*) [10], 2-succinyl-6-hydroxy-2,4-cyclohexadiene-1-carboxylic acid synthase/2-oxoglutarate decarboxylase (*menD*) [9], and cytochrome oxidase (*ctaA*) [11]. In clinical strains the genetic basis of SCV phenotypes has been reported in only a few cases. In thymidine-auxotrophic SCVs from cystic fibrosis patients, mutations were identified in the thymidylate synthase gene, *tyhA* [12], [13] and in menadione-auxotrophic SCVs collected from patients with chronic osteomyelitis the mutations causing the SCV phenotype were localized to *menB*, the gene encoding naphthoate synthase [14].

Fusidic acid (FA) is an antibiotic used as a single agent or in combination therapies for the treatment of *S. aureus* infections. FA acts by binding to the translation elongation factor EF-G when in complex with the ribosome during translation. FA binding prevents the release of EF-G from this complex and interrupts further protein synthesis. In *S. aureus* several genetically distinct classes of FA-resistance mechanisms have been identified. The FusA class results from mutations within the gene *fusA* which encodes EF-G thereby affecting EF-G structure and fusidic acid-target binding [15], [16], [17], [18]. A subset of FusA mutants display the SCV phenotype and are auxotrophic for hemin [18]. The FusB, FusC and FusD classes carry fusidic acid resistance genes which can be horizontally-transmitted and produce proteins that protect EF-G from fusidic acid binding [19], [20], [21], [22], [23]. The FusE class of mutants, all of which display the SCV phenotype, harbour knockout-mutations (mutations causing frameshifts, nonsense codons, and mutated start codons) in *rplF*, the gene coding for ribosomal protein L6 [18]. Note that fusidic acid-resistant mutants with the SCV phenotype can be selected either with fusidic acid or with aminoglycosides such as kanamycin [18]. Some FusE mutants are auxotrophic for hemin, while others are auxotrophic for menadione [18]. The phenotypic variation within the FusE-group cannot be explained by the *rplF* mutations alone, since mutants with identical mutations in *rplF* can display either hemin or menadione auxotrophy [18]. This implies that there must be additional mutations in some, or all, of the FusE mutant strains.

In the present study the mutational status of the FusE fusidic acid-resistant mutant group is characterized, and we show that this group encompasses three genetically distinct subgroups, all of which harbour *rplF* mutations. Two of these subgroups carry additional mutations in genes associated with hemin or menadione biosynthesis, which can explain their hemin or menadione auxotrophy. We also report on the selection and identification of mutations that compensate for the slow growth phenotype of FusA and FusE SCV's.

## Results

### Genetic analysis of FusE class mutants

FusE mutants are FA-resistant, and harbour mutations in *rplF* [18]. FusE mutants have also been placed in phenotypic subclasses depending on whether they are auxotrophic for hemin or menadione. Different FusE mutants demonstrate significantly different generation times in LB of 50–200 min compared to the AH001 WT parent strain (24 min) [18]. Note that the growth rate of all of the SCV's can be rescued by the addition of hemin or menadione, as appropriate, to the growth media [18]. These differences in auxotrophy and growth rate suggested that at least some FusE mutants carry additional mutations that lead to the unique phenotypes observed. 23 of the 24 partially characterized FusE mutants [18] were chosen for this investigation (Table 1). Only the slowest growing FusE mutant, strain AH330 [18], was dropped from this investigation due to its extreme phenotypic instability. Because the SCV phenotype in *S. aureus* has been linked to defects in hemin and menadione biosynthesis all known genes (Table S1) predicted to be involved in these pathways were PCR-amplified and sequenced for the set of mutants investigated in this study, according to their observed auxotrophy (Table 1). We also re-sequenced *rplF* in each mutant to confirm the presence of the expected mutation. In 11 of 15 hemin-auxotrophic FusE mutants (AH380 to AH357, Table 1), the original *rplF* mutations [18] were confirmed, and no additional mutations were identified in the gene set sequenced (Table S1). The *rplF* mutations in the remaining 4 hemin-auxotrophic FusE mutant strains (AH374 to AH384, Table 1) were confirmed and in addition, mutations were also identified in *hemA*, *hemB* or *hemH*. All 4 *hem* mutations caused single amino acid substitutions.

**Table 1:** Genetics of FusE class mutants.

	Identified mutations		
Strain	*fusA*	*rplF*	Men/Hem gene^a^
**FusE L6**			
AH380	WT	−1 nt 158 (FS^b^)	none
AH352	WT	−1 nt 106 (FS)	none
AH377	WT	+1 nt 23 (FS)	none
AH348	WT	TGA stop nt 244	none
AH370	WT	TAA stop nt 229	none
AH346	WT	−1 nt 238 (FS)	none
AH333	WT	TAA stop nt 249	none
AH392	WT	−1 nt 383 (FS)	none
AH379	WT	TAA stop nt 418	none
AH349	WT	−1 nt 404 (FS)	none
AH357^c^	WT	TAA stop nt 301	none
**FusE L6 Hem**			
AH374	WT	−5 nts 236−240 (FS)	*hemA* Ala190Glu
AH339	WT	−1 nt 144 (FS)	*hemB* Gly87Asp
AH337	WT	−1 nt 220 (FS)	*hemB* Gly87Asp
AH384	WT	−1 nt 221 (FS)	*hemH* Gly209Asp
**FusE L6 Men**			
AH378	WT	−1 nt 221 (FS)	*menB* Arg101Leu
AH351	WT	−4 nts 45–48 (FS)	*gerC3* −1 FS nt 919
AH358^b^	WT	−1 nt 423 (FS)	*menB* Val58Phe
AH360	WT	ATA Start nt 3	*menA* Arg19Ser
AH383	WT	TAA stop nt 139	*menA* Gly87Asp
AH362	WT	TAA stop nt 282	*menA* Asp81Tyr
AH390	WT	+1 FS nt 84	*menB* Arg 53 Cys
AH386	WT	−1 FS nt 90	*menB* Gln 204 stop

a All genes known to be involved in hemin and menadione biosynthesis were sequenced in the respective groups to identify mutations, see Table S1.

b Mutation is predicted to cause a ribosomal frameshift (FS) during translation of the mRNA.

c AH357 and AH358 were also analysed by CGS in comparison with wild-type 8325-4.

The *rplF* mutations in all 8 menadione-auxotrophic mutants (AH378 to AH386, Table 1) were confirmed [18] and in addition mutations were identified in *menA*, *menB* or *gerC3* in these mutants. Within this group of strains we now place AH358 which was previously mistakenly listed as a hemin auxotroph [18]. The mutations associated with menadione-auxotrophy caused single amino acid substitutions, with the exception of a frameshift mutation in *gerC3* in strain AH351 and a nonsense codon in *menB* in strain AH386, both near the end of the respective gene. The absence of randomly distributed frameshift or nonsense mutations in these genes in this strain group raises the possibility that the mutant proteins may retain some activity. Not finding a mutation in a suspect gene does not rule out the possibility that mutations at other sites could be responsible for the phenotype. To test whether we had identified all mutations relevant to the phenotypes, we arbitrarily chose two mutant strains (AH357, AH358) for comparative genome sequencing (CGS, see Materials and Methods for details) and comparison to parent strain AH001 (Table 1). CGS confirmed the expected genotypes of each mutant and did not reveal the presence of any additional mutations (additional SNPs were called by CGS but ruled out as false positives after local re-sequencing; see Materials and Methods). While we cannot rule out the possibility that there may be additional mutations in strains that were not analysed by CGS, for those strains that were tested the results were in agreement with that from sequence analysis of the listed suspect genes affecting hemin and menadione biosynthesis (Table S1).

Thus, our data now demonstrate that the FusE class of mutants comprise three sub-groups: the FusE L6 class (*rplF* single mutants), the FusE L6-Hem class (*rplF* and *hem* double mutants) and the FusE L6-Men class (*rplF* and *men* double mutants). Based on these data we hypothesized that the phenotypes of FusA SCV [18] and FusE L6 mutants could be explained by single mutations in *fusA* or *rplF*, respectively. Further, that the phenotypes of FusE L6-Hem and FusE L6-Men mutants could each be explained by the presence of at least two mutations, one affecting *rplF* and another in a gene involved in either hemin or menadione biosynthesis, respectively. If this hypothesis is correct, it should be possible to reverse the mutant phenotypes by selection of second-site compensatory mutations in each of the affected genes. To test this we performed a reversion analysis by selecting growth-compensated mutants of strains belonging to each of the FA-resistant SCV classes (FusA SCV, FusE L6, FusE L6-Hem, and FusE L6-Men).

### Analysis of growth-compensated FusA SCV mutants

FusA SCVs are caused by mutations in elongation factor EF.G. From the FusA SCV group [18], independent growth compensated mutants were selected from four different strains (AH347, AH350, AH364 and AH366, Table 2) as described in Materials and Methods. In all mutants the *fusA* gene was sequenced to identify possible compensatory mutations that may have occurred in these fast-growing derivatives. In most cases intragenic second-site mutations were identified in *fusA* (Table 2), the only exception being strain AH537 where the compensatory mutation was a reversion back to the WT *fusA* sequence. Changes in KAN and FA MICs were also measured. Most compensatory mutants had decreased KAN and FA MIC's compared to the parent strain, although it remained higher than the original WT strain NCTC 8325-4 MIC (Table 2). This is in agreement with published data on the MICs associated with intragenic mutations in *fusA* [15] and suggests that the double mutants do not fully restore a wild-type EF-G phenotype. The only strain in which MIC was identical to that of the wild-type was AH537 in which the *fusA* mutation had reverted to the wild-type sequence. In a few of the mutants the compensatory *fusA* mutation actually led to a small increase in FA MIC (AH519, AH521, AH530, AH539 and AH541) or KAN MIC (AH527, and AH541). This finding is of interest since these mutants were not selected in the presence of fusidic acid or kanamycin. Mutant AH540 did not have a *fusA* compensatory mutation and displayed increased FA and KAN MICs compared to parent strain AH366 (Table 2). Therefore the unknown extragenic growth-compensatory mutation(s) in this mutant directly affects resistance levels to these two antibiotics. Because we have not sequenced the complete genomes for any of these mutants we cannot rule out the possibility that additional compensatory mutations might have been selected. Collectively, these observations demonstrate that growth compensation in the FusA class mutants is strongly associated with second-site mutations in *fusA*.

**Table 2:** Growth compensated mutants of FusA SCVs.

Stock	SCV mutation	Compensating mutation	MIC (µg/ml)		
	*fusA* (EF-G)	*fusA* (EF-G)	KAN	FA	N^a^
**AH001**	WT	WT	2	0.064	
**AH347**	Arg659Ser		48	12	
AH517	Arg659Ser	Gly653Ala	12	4	2
AH518	Arg659Ser	Val86Ile	12	8	
AH519	Arg659Ser	Val86Leu	12	16	
AH520	Arg659Ser	Pro635ProPro	12	4	
AH521	Arg659Ser	Ala478Val	32	16	
AH523	Arg659Ser	Gly653Cys	6	3	
**AH350**	Arg659Cys		12	12	
AH524	Arg659Cys	Val86Leu	8	8	2
AH525	Arg659Cys	Val86Ile	8	4	3
AH527	Arg659Cys	Ala217Thr	32	6	
AH530	Arg659Cys	Ala376Val	12	16	
AH531	Arg659Cys	Met616Ile	12	4	
**AH364**	Asp434Asn		24	256	
AH532	Asp434Asn	Val86Ile	12	256	2
AH534	Asp434Asn	Ala376Val	12	256	
AH535	Asp434Asn	Ala655Val	6	8	
**AH366**	Pro114His		12	6	
AH536	Pro114His	Gly653Val	6	0.25	2
AH537	Reversion	WT	2	0.094	
AH538	Pro114His	Val86Ile	8	2	2
AH539	Pro114His	Ser416Tyr	12	24	
AH540	Pro114His	Extragenic^b^	24	8	
AH541	Pro114His	Ile408Thr	16	16	
AH542	Pro114His	Gly653Ala	4	1.5	
AH544	Pro114His	Gly653Cys	4	0.75	

a N, the number of independent isolates with the same genotype and phenotype.

b No compensatory mutation was identified within *fusA*.

### Analysis of a menadione-auxotrophic FusA SCV mutant

We previously identified an unusual FusA-SCV mutant (AH334) that carried a mutation in *fusA* but also displayed menadione-auxotrophy [18]. Sequence analysis of AH334 (CGS and local sequencing) showed that in addition to a mutation in *fusA* this mutant also carried a TGA nonsense mutation in *menE*, thus providing a plausible explanation for the menadione-auxotrophy (Table 3). Growth-compensated mutants were selected from AH334 as described above for the other FusA-SCV mutants. The genomes of our WT strain AH001 (NCTC 8325-4), the menadione-auxotrophic FusA-SCV mutant (AH334), and one of 4 independently selected growth-compensated mutants (AH513) were each analysed by CGS. The DNA sequence showed that the growth-compensated mutant AH513 had acquired a mutation (C→T substitution) 60 nucleotides upstream of the start codon of *menE* (Table 3). By comparison with other *S. aureus* sequences we drew the tentative conclusion that this mutation altered the −35 region of the *menE* promoter. If this conclusion were correct it would suggest the possibility that an up-regulation of the mutant *menE* gene may suppress the defect in menadione biosynthesis and thus improve growth rate. Sequencing of the *menE* region of the 3 additional growth compensated mutants revealed that one of these (AH515) had acquired the same mutation at −60 (Table 3). No additional mutations in the *menE* region were identified in the other two mutants. As with the growth compensated FusA SCV mutants discussed above, the growth compensatory mutations once again altered FA and KAN MICS. All 4 growth-compensatory mutants of parent strain AH334 demonstrated reduced MICs to both of these drugs (Table 3).

**Table 3:** Growth compensated mutants of a FusA menadione-auxotrophic SCV.

Strain	SCV mutations		Compensating mutation	MIC (µg/ml)	
	*fusA*	*menE*	*menE*	KAN	FA
AH001^a^	WT	WT		2	0.064
AH334^a^	Thr436Ile	Trp67Stop		128	48
AH513^a^	Thr436Ile	Trp67Stop	CT (−60)	16-34	12-16
AH514	Thr436Ile	Trp67Stop	none found.	16-34	12-16
AH515	Thr436Ile	Trp67Stop	CT (−60)	16-34	12-16
AH516	Thr436Ile	Trp67Stop	none found	16-34	12-16

a AH001 (8325-4), AH334, and AH513 were also analysed by CGS.

### Analysis of growth-compensated FusE L6 mutants

In the FusE L6 group we predicted that mutations in *rplF* cause the slow growth phenotype. These strains are phenotypically stable and faster-growing mutants did not arise at a frequency high enough to be consistently isolated by spreading∼10^8^ cfu on LA-plates. Instead, strains AH379, AH380 and AH377 were passaged twice in LB overnight to enrich for rare faster-growing mutants of each strain (Table 4). The strains used in this selection had putative knockout-mutations in *rplF* in the form of a stop codon (AH379), a −1 nt frameshift (AH380) and a +1 nt frameshift (AH377). In 16 growth-compensated mutants isolated from independent selections (Table 4), these interruptions in *rplF* had been reversed by nt substitutions in the stop codon (AH379-derived mutants), downstream +1 nt frameshifts (AH380-derived mutants) and overlapping or downstream −1 nt frameshifts (AH377-derived mutants). In two of the AH377-derived mutants (AH588 and AH589) the acquisition of the −1 frameshift mutation was associated with the simultaneous acquisition of additional base substitution mutations in an AT-rich sequence nearby in *rplF* (Table 4). Interestingly, all of the selected FusE L6 growth-compensated mutants appear to be full phenotypic revertants with respect to both colony size and FA and KAN MICs (Table 4). We have not sequenced the genomes of these mutants and thus cannot formally rule out the possibility that they carry additional mutations associated either with the SCV or with the selection for growth compensation. However, the full phenotypic compensation noted above supports the hypothesis that the *rplF* mutations alone can explain the phenotypes of slow growth and increased FA and KAN MICs in the FusE L6 group.

**Table 4:** Selection of growth-compensated mutants from FusE L6 SCVs.

	Mutations in *rplF* (ribosomal protein L6)				
Strain	SCV mutation	Compensating mutation	MIC (**µ**g/mL)		N^a^
			KAN	FA	
AH001 (WT)			2	0.064	
AH379	Gln140Stop (CAA→TAA)		8	6	
AH592	replaced	TAA→GAA (Glu)	2	0.064	2
AH593	replaced	TAA→TTA (Leu)	3	0.047	2
AH594	replaced	TAA→AAA (Lys)	3	0.064	
AH595	replaced	TAA→TAT(Tyr)	3	0.064	3
AH380	−1 T nt 158		8	6	
AH597	−1 T nt 158	+T nt 170	3	0.064	2
AH598	−1 T nt 158	+C nt 173	3	0.047	
AH600	−1 T nt 158	+T nt 176	2	0.032	
AH377	+A nt 23, KKIID→KKNYstop		8	2	
AH588^b^	+A nt 23	−G nt 18 →NKIFD	3	0.064	
AH589^b^	WT	−A nt 23 →KKIFD	3	0.064	
AH590^b^	+A nt 23	−G nt 18 →KKIID WT	2	0.032	2

a N, the number of independent isolates with the same genotype and phenotype.

b Sequence complexity of AH377 derivatives.

WT: AAG-AAA-ATT-ATT-GAC (KKIID).

AH377: AAG-AAA-A**A**T-TAT-TGA (KKNYstop).

AH588: AA**T**-AAA-**A**TT-**T**TT-GAC (NKIFD).

AH589: AAG-AAA-ATT-**T**TT-GAC (KKIFD), and Glu114Leu (GAA→TTA).

AG590: AAA-AAA-**A**TT-ATT-GAC (KKIID).

### Analysis of growth-compensated FusE L6-Hem and L6-Men mutants

28 independent growth-compensated mutants were selected from 6 mutant strains (AH339, AH384, AH386, AH362, AH351 and AH390) belonging to the FusE L6-Hem and FusE L6-Men groups (Table 5). In all cases, the original *rplF* mutation of the respective parental strain was still present in the compensated strains (Table 5). In 3 growth-compensated mutants the original *hem* or *men* mutation had reverted to the WT sequence (AH559, *hemB →* WT; AH483 and AH488, *menB* → WT) (Table 5). In 4 growth-compensated mutants a second mutation had occurred in *menB*: in strains AH484, AH485, and AH486, by replacement of a nonsense codon with a sense codon; and in AH492, by the acquisition of a second-site amino acid substitution (Table 5). One of the growth-compensated mutants selected from AH351 (*gerC3*) had acquired a single nt substitution in the predicted promoter region upstream of the operon containing *gerC* (Table 5). This suggests the possibility that an up-regulation of the expression of the whole operon (which includes the genes *gerC1*, *ubiE* and *gerC3*) can compensate for the reduced activity of the enzyme-component encoded by the mutant *gerC3*. In many growth-compensated mutants intragenic mutations were not identified by sequencing a set of genes believed to be involved in hemin or menadione biosynthesis (Table S1) suggesting that in some cases compensatory mutations can arise in other genes.

**Table 5:** Selection of growth-compensated mutants from FusE Hemin and Menadione-auxotrophic SCVs.

	Identified mutations				
		Mutations in *men* or *hem* genes		MIC (µg/ml)	
Strain	*rplF* (L6)^a^	Original	Intragenic	KAN	FA
**AH001 (WT)^b^**				2	0.064
**AH339**	−1 FS^c^ nt 144	*hemB* Gly87Asp		256	8
AH559	−1 FS nt 144	replaced	*hemB* WT	12	4
**AH384**	−1 FS nt 221	*hemH* Gly209Asp		128	12
AH497^b^	−1 FS nt 221	*hemH* Gly209Asp	NMI^d^	64	8
AH498	−1 FS nt 221	*hemH* Gly209Asp	NMI	64	12
AH499	−1 FS nt 221	*hemH* Gly209Asp	NMI	48	12
AH501	−1 FS nt 221	*hemH* Gly209Asp	NMI	48	8
AH502	−1 FS nt 221	*hemH* Gly209Asp	NMI	64	8
AH503	−1 FS nt 221	*hemH* Gly209Asp	NMI	48	6
AH510	−1 FS nt 221	*hemH* Gly209Asp	NMI	96	6
**AH386**	−1 FS nt 90	*menB* Gln204stop		256	12
AH482	−1 FS nt 90	*menB* Gln204stop	NMI	48	12
AH483	−1 FS nt 90	replaced	*menB* WT	16	3
AH484	−1 FS nt 90	replaced	*menB* 204Ser	24	8
AH485	−1 FS nt 90	replaced	*menB* 204Lys	16	6
AH486	−1 FS nt 90	replaced	*menB* 204Lys	12	6
AH487	−1 FS nt 90	*menB* Gln204stop	NMI	24	8
AH488	−1 FS nt 90	replaced	*menB* WT	24	8
**AH362**	TAA stop nt 282	*menA* Asp81Tyr		256	16
AH479	TAA stop nt 282	*menA* Asp81Tyr	NMI	256	16
AH481	TAA stop nt 282	*menA* Asp81Tyr	NMI	192	4
**AH351**	−4 FS nts 45–48	*gerC3* −1 FS nt 919		128	12
AH472	−4 FS nts 45–48	*gerC3* −1 FS nt 919	NMI	24	8
AH473	−4 FS nts 45–48	*gerC3* −1 FS nt 919	NMI	32	8
AH474	−4 FS nts 45–48	*gerC3* −1 FS nt 919	NMI	32	6
AH475	−4 FS nts 45–48	*gerC3* −1 FS nt 919	NMI	64	12
AH476	−4 FS nts 45–48	*gerC3* −1 FS nt 919	NMI	16	6
AH477	−4 FS nts 45–48	*gerC3* −1 FS nt 919	NMI	16	16
AH478	−4 FS nts 45–48	*gerC3* −1 FS nt 919	*gerC* T→G at −59	48	16
**AH390**	+1 FS nt 84	*menB* Arg53Cys		256	12
AH489	+1 FS nt 84	*menB* Arg53Cys	NMI	32	6
AH490	+1 FS nt 84	*menB* Arg53Cys	NMI	48	6
AH491	+1 FS nt 84	*menB* Arg53Cys	NMI	64	16
AH492	+1 FS nt 84	*menB* Arg53Cys	*menB* Val128Ile	24	6

a Note that the *rplF* mutations were published previously [18].

b AH001 (W.T., 8325-4) and AH497 were also analysed by CGS.

c FS, frameshift mutation.

d NMI. no mutation identified.

### Influence of hemin or menadione addition on KAN MIC

**Figure 1:**
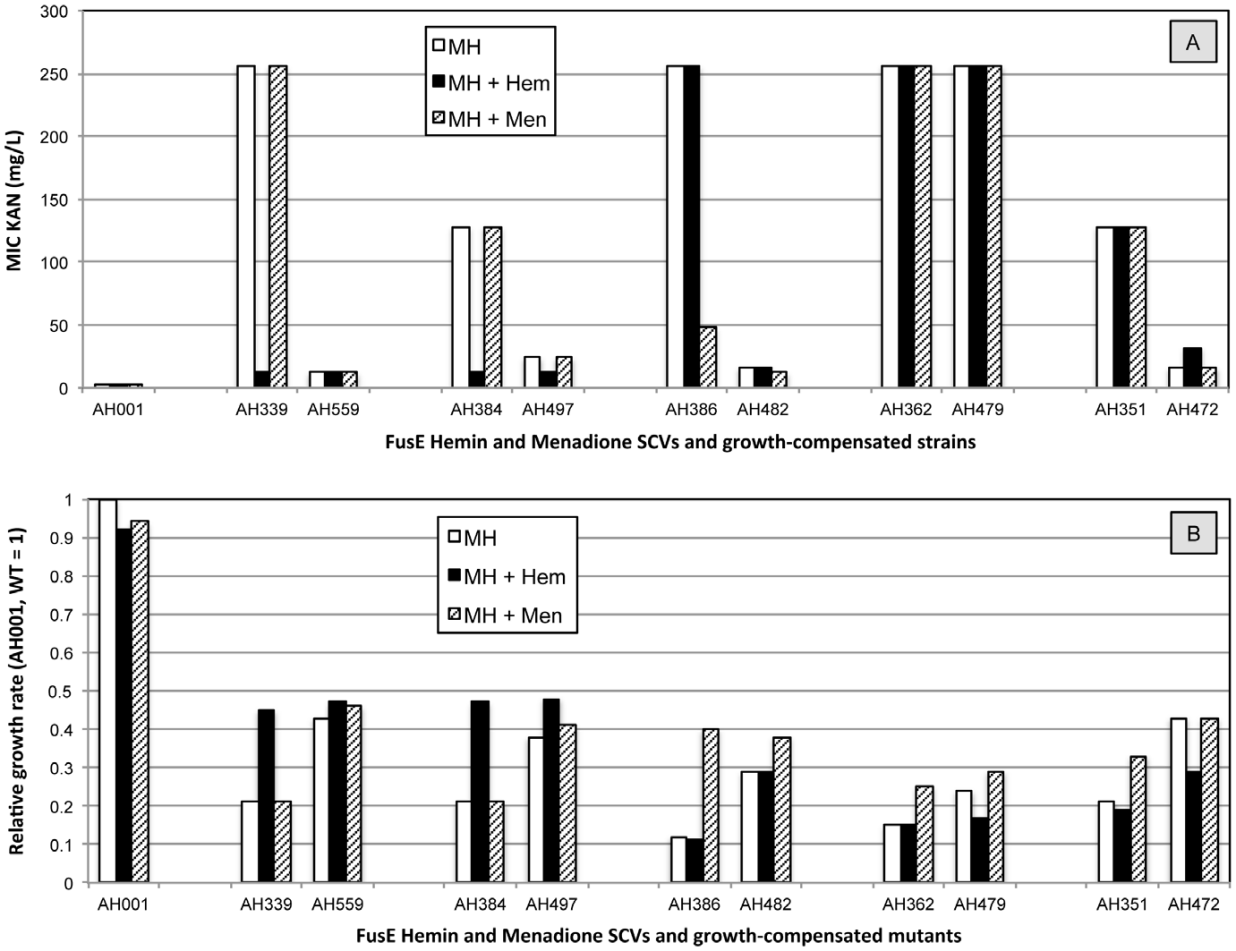
The affect of the addition of hemin or menadione to the growth medium (Mueller-Hinton Broth or agar) on MIC KAN (part A) and growth rate (part B) for isogenic strain pairs (SCVs and growth-compensated mutants). AH001 is the wild-type strain. Results shown are the median value of six to ten independent measurements for MIC measured by Etest, and the mean of two independent measurements of growth rate measured in a Bioscreen C machine.

The data show that the KAN MIC's of growth-compensated FusE SCV's are almost always intermediate between the high MIC values of the parental SCV strain and the low, fully susceptible, MIC value of the wild-type (Table 5). The reduced susceptibility of hemin and menadione auxotrophic SCVs to aminoglycoside antibiotics like KAN is thought to be associated with defects in the proton motive force which is required for aminoglycoside uptake [1]. We chose a representative set of the FusE SCV mutants with identified mutations in *hemB*, *hemH*, *menA*, *menB* and *gerC* (Table 5) asked whether their KAN susceptibility or growth rates could be complemented by the addition of hemin or menadione to the growth medium. In each case we compared the original SCV mutant with an isogenic growth-compensated mutant (Figure 1). The addition of hemin, but not menadione, caused a large reduction in the KAN MIC of the hemin auxotrophic SCVs (AH339 and AH384) whereas is had no significant effect on the MICs of isogenic growth-compensated mutants (AH559 and AH497) (Figure 1A). The growth rate of the *hemB* and *hemH* mutant strains was also increased by the addition of hemin to the growth medium, to the same rate as that associated with the selected growth-compensatory mutations in AH559 and AH497 (Figure 1B). The addition of menadione did not alter the growth rate of these strains. The addition of menadione, but not hemin, caused a large reduction in the KAN MIC of the *menB* auxotroph AH386 (and AH390, not shown in Figure 1) but did not alter the MIC of AH362 (*menA*) or AH351 (*gerC*). We note that the KAN MIC associated with the *menA* mutation in AH362 was also not reduced in the isogenic growth-compensated mutant AH479. The addition of menadione, but not hemin, increased the growth rates of each of the menadione-auxotrophic strains to a similar rate as that achieved by the growth-compensatory mutations (Figure 1B). We note here that the degree of growth compensation, whether by compensatory mutations affecting *hem* or *men* biosynthesis genes, or by chemical complementation of auxotrophy through the addition of hemin or menadione to the growth medium, is in all cases incomplete (Figure 1B). This suggests that in these strains the *rplF* mutations inactivating ribosomal protein L6 are alone sufficient to cause a severe growth defect. This is in agreement with published data [18] showing that the SCV strains carrying only a mutation in *rplF* (such as AH357, analysed by CGS, Table 1) can have growth rates less than half that of the wild-type strain. Chemical complementation experiments were also made with FusA and FusE L6 mutants but in those strains the addition of hemin or menadione caused no change in MIC KAN.

### Two-step genetic reversion of an SCV phenotype

**Figure 2:**
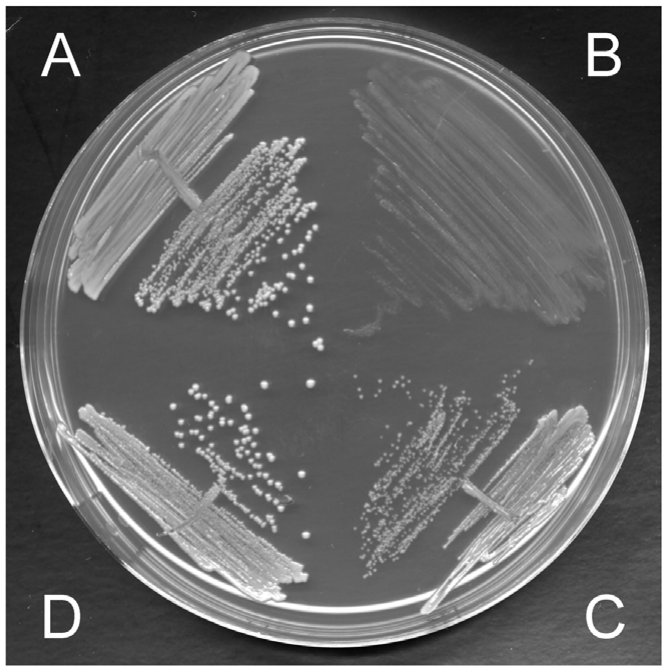
Illustration of a FusE L6-Men SCV derived from *S. aureus* 8325-4, and how it was evolved back to normal growth by selecting for faster-growing mutants in two steps (clockwise). A. *S. aureus* AH001 (WT), normal growth; B. AH386, FusE L6-Men SCV (*rplF, menB*); C. AH483 (*rplF*, *menB* WT); D. AH562 normal growth (*rplF* WT, *menB* WT).

DNA sequence analysis (CGS and local sequencing) supports the hypothesis that the slow growth phenotype of the FusE L6-Hem and FusE L6-Men SCV mutants can be explained by the mutations identified in *rplF* and *hem/men* genes (Table 5). If this hypothesis were correct it would predict that it should be possible to select a WT phenotype from such an SCV in two steps, by reverting first one mutation, then the second. To test this we selected growth-compensatory mutations from AH386 (Table 1). AH386 is extremely slow growing menadione-auxotrophic SCV (generation time 180 min in LB medium) and carries mutations in *rplF* and *menB*. In the first-step selection for a faster-growing mutant we isolated AH483. In AH483 the *menB* sequence has reverted to the WT sequence (Gln204) and this was associated with a reduction in the FA and KAN MICs (Table 6). AH483 was cycled in five independent liquid overnight cultures for two days, and two of these cultures were taken over by faster-growing cells that were isolated as the independent mutants AH562 and AH563 that were subsequently characterized (Table 6). In AH562 the *rplF* frameshift had reverted to the WT sequence (+A), while in AH563 a +G insertion in the same position had reverted the original frameshift and replaced it with a silent mutation. Both strains displayed colony sizes and MIC's for FA and KAN that were indistinguishable from the WT strain *S. aureus* 8325-4 (Figure 2). The ability to revert the SCV phenotype in two steps supports the hypothesis that the SCV phenotype in AH386 can be explained by the two mutations identified in *rplF* and *menB*, although in the absence of further experiments we cannot conclusively rule out the possibility that additional mutations might also be involved.

**Table 6:** Selection of growth-compensated mutants from an SCV in two steps.

	SCV mutations		MIC (µg/ml)	
Strain	*rplF*	*menB*	KAN	FA
AH001	WT	WT	2	0.064
AH386	−A nt 90	Gln204UAG (Stop)	256	12
AH483	−A nt 90	Stop204Gln (WT)	16	3
AH562	+A nt 90 Lys (AAA = WT)	WT	2	0.064
AH563	+G nt 90 Lys (AAG)	WT	2	0.064

## Discussion

The FusE class of SCV's have reduced susceptibility to FA and aminoglycosides and carry putative knockout-mutations (frameshifts, nonsense codons, or mutated start codons) in *rplF*, the gene coding for ribosomal protein L6. We showed previously that FusE mutants comprised at least two distinct phenotypic groups, being auxotrophic for either hemin, or menadione [18]. In addition, different FusE mutants displayed large variations in growth rate as well as in phenotypic stability. This suggested that at least some FusE mutants must carry additional mutations to explain the diversity in phenotypes. Here we have shown that FusE mutants fall into three distinct genetic and phenotypic classes: the FusE L6 class (*rplF* single mutants, slow growing), the FusE L6-Hem class (*rplF* and *hem*-gene double mutants, very slow growing) and the FusE-Men class (*rplF* and *men*-gene double mutants, extremely slow growing). CGS, localized manual sequencing, and genetic reversion studies all support the conclusion that many of the FusE SCV's are double mutants. FusA-SCV mutants in contrast typically carry only single mutations in *fusA*, but one unusual variant was identified that carried an additional mutation in the menadione biosynthesis pathway.

Because we have performed CGS on only a very limited number of strains, we cannot rule out the possibility that additional unidentified mutations may be present in some of our strains and contribute to their phenotypes.

In order to link the identified mutations in FusE and FusA-SCV strains to the phenotypes of the respective groups we selected growth-compensated mutants that were subjected to genetic analysis. For each of the different FusE classes we found that reversion or intragenic mutation led to the predicted improvement in growth. In one FusE mutant we made a two-step selection and identified reversion of a single primary mutation at each step (Table 6). The FusE mutants identified as double mutants (Table 1) were selected using the aminoglycoside kanamycin. It seems unlikely that the double mutations pre-existed in the overnight cultures, especially at frequencies similar to the *fusA* and *rplF* single mutants. More likely is that they are derived from mutants with single (*rplF* or *hem*/*men*) mutations and that secondary mutations arising under selection improved resistance or survival in the presence of kanamycin. Although we identified second-site mutations and reversions in *hem* and *men* genes when selecting for growth compensation of SCVs, these were not always found. This suggests that there are additional ways in which the SCV phenotype can be ameliorated (some of these are under investigation). We note that the level of resistance to KAN measured in the FusE SCVs (Table 5) appears to be partly associated with the respiratory defects caused by mutations in *hem* or *men* genes, and partly by the mutation in ribosomal protein *rplF*. Thus, the addition of hemin or menadione to the growth medium reduces the KAN MIC of these double mutants to a level similar to that associated with the *rplF* mutation alone (Figure 1 and Table 4).

These data show that even in the controlled laboratory environment, a simple selection for resistance to one antibiotic frequently result in the selection of different mutant variants, some of which have complex mutant phenotypes and genotypes. Thus, aminoglycosides selected different phenotypic variants of *S. aureus* including those that were SCVs and were resistant to both aminoglycosides and to the unrelated antibiotic fusidic acid. Among these mutants, FusE class isolates carried mutations in one or two of 6 different genes (*rplF*, *hemB, hemH, menA, menB, genC*). Many of these resistant isolates had very slow growth rates in rich medium and under selection for faster growth the typically evolved by acquiring additional mutations. This mode of compensatory evolution will act to fix some or all of the original mutations in these isolates. While many of the SCV mutants initially selected for resistance may be removed by natural selection for growth rate, many of those that survive can be expected to undergo further evolution, with the accumulation of additional mutations, some associated with selection for resistance and other associated with selection for restoration of growth or survival fitness.

## Materials and Methods

### Strains and growth conditions

FusA SCV and FusE SCV mutants were selected from the drug-susceptible laboratory wild-type (WT) strain *S. aureus* 8325-4 (AH001), as previously described [18]. Bacterial cultures were grown in Luria broth (LB), or on solid Luria agar plates (LA) unless otherwise stated. Liquid cultures were incubated overnight (18 h) at 37°C with shaking at 200 rpm. Bacterial strain stocks were stored frozen in LB + DMSO (7%) at −80°C. Maximum exponential growth rates were measured in a Bioscreen C (OY Growth Systems, Finland) and are based on 6–8 independent measurements for each strain and condition.

### Selection of fast-growing compensated mutants or revertants from SCVs

Independent SCV colonies from various strains were suspended in 0.9% NaCl, diluted, plated on LA and visually inspected for faster-growing colonies following overnight growth (37°C). Colonies were then picked, restreaked, and stocked. DNA sequencing was used to determine if these faster-growing mutants had acquired intragenic compensatory mutations or reversions in any of the mutations associated with the SCV phenotype of the strain.

### MIC assays

The minimal inhibitory concentration was measured using Etest strips (BioMérieux) in accordance with the manufacturers' recommendations. Initially, a single colony was dissolved in 1 ml 0.9% NaCl to a density of 0.5 McFarland units, and a cotton swab was used to spread the bacterial suspension over the surface of a Mueller-Hinton plate (Difco Becton Dickinson, MD, USA). After the application of the Etest strip, plates were incubated at 37°C for 18–24 h until growth of the bacterial lawn was evident.

### PCR and DNA sequencing

Template DNA was prepared by initially dissolving a bacterial colony in 100 µl ddH2O and placing this cell suspension in a microfuge tube. Next,∼100 µl of 0.25 mm acid washed glass beads (Sigma-Aldrich AB, Stockholm, Sweden, cat no. G1277) was added to the cell suspensions which were then vortexed violently for 10 sec to disrupt the cell walls. Primers for amplifying and sequencing *S. aureus* genes were designed based on the publicly available genome sequences. The standard PCR sequence for our gene amplifications began with a denaturation step of 5 min at 95°C, followed by 30 cycles of 15 sec at 95°C, 15 sec at 45°C and 1 min/kb 72°C. The last elongation step was prolonged to 5 min. Sequencing reactions were carried out at Macrogen Inc. (Seoul, Korea) and sequences were then analyzed using Vector NTI 10.3.0 (Invitrogen, Carlsbad, CA) and BioEdit (http://www.mbio.ncsu.edu/BioEdit/bioedit.html), a freely available sequence alignment editor. Twelve different genes associated with hemin biosynthesis, including their predicted promoter regions, were sequenced in all hemin-auxotrophic SCV strains (Table S1). Nine different genes associated with menadione biosynthesis, including their predicted promoter regions, were sequenced in all menadione-auxotrophic SCV strains (Table S1).

### Comparative genome sequencing

CGS services provided by Roche NimbleGen Inc. (Madison, WI, USA) were utilized for whole genome mutation mapping comparing parent strain AH001 (NCTC 8325-4) with fusidic acid-resistant SCV NCTC 8325-4 mutants. A *S. aureus* NCTC 8325 genome (Genbank accession: CP000253.1) tiling array was used to hybridize test and reference genomic DNA, and single nucleotide polymorphisms in each strain were identified based on previously defined criteria [24]. The number of SNP's called by CGS was up to 30 per genome sequence. Each of these putative mutations was subjected to manual re-sequencing of the relevant genes and the great majority (with the exception of those named in this paper) were eliminated as false positives. The SNP's called by CGS included all of those expected to be present in each genome on the basis of prior manual sequencing, with the exception of one frameshift mutation in *rplF* (AH358) which was not called but was confirmed by manual re-sequencing.

## Supporting Information

Table S1 The following genes including predicted promoter regions (given here with locus tags and product names from the NCTC 8325 genome, GenBank accession number CP000253.1, available at http://www.ncbi.nlm.nih.gov/), were sequenced in all menadione-auxotrophic or hemin-auxotrophic SCV strains. If gene names were not annotated in NCTC 8325 then the names were taken from a closely related *S. aureus* genome, usually *S. aureus* COL (GenBank: CP000046.1, available at http://www.ncbi.nlm.nih.gov/).
(DOCX)

## References

[1] Proctor RA, von Eiff C, Kahl BC, Becker K, McNamara P (2006). Small colony variants: a pathogenic form of bacteria that facilitates persistent and recurrent infections.. Nat Rev Microbiol.

[2] Balwit JM, van Langevelde P, Vann JM, Proctor RA (1994). Gentamicin-resistant menadione and hemin auxotrophic Staphylococcus aureus persist within cultured endothelial cells.. J Infect Dis.

[3] Jonsson IM, von Eiff C, Proctor RA, Peters G, Ryden C (2003). Virulence of a hemB mutant displaying the phenotype of a Staphylococcus aureus small colony variant in a murine model of septic arthritis.. Microb Pathog.

[4] Proctor RA, van Langevelde P, Kristjansson M, Maslow JN, Arbeit RD (1995). Persistent and relapsing infections associated with small-colony variants of Staphylococcus aureus.. Clin Infect Dis.

[5] von Eiff C, McNamara P, Becker K, Bates D, Lei XH (2006). Phenotype microarray profiling of Staphylococcus aureus menD and hemB mutants with the small-colony-variant phenotype.. J Bacteriol.

[6] von Eiff C, Peters G, Becker K (2006). The small colony variant (SCV) concept – the role of staphylococcal SCVs in persistent infections.. Injury.

[7] Chuard C, Vaudaux PE, Proctor RA, Lew DP (1997). Decreased susceptibility to antibiotic killing of a stable small colony variant of Staphylococcus aureus in fluid phase and on fibronectin-coated surfaces.. J Antimicrob Chemother.

[8] von Eiff C, Heilmann C, Proctor RA, Woltz C, Peters G (1997). A site-directed Staphylococcus aureus hemB mutant is a small-colony variant which persists intracellularly.. J Bacteriol.

[9] Bates DM, von Eiff C, McNamara PJ, Peters G, Yeaman MR (2003). Staphylococcus aureus menD and hemB mutants are as infective as the parent strains, but the menadione biosynthetic mutant persists within the kidney.. J Infect Dis.

[10] Schaaff F, Bierbaum G, Baumert N, Bartmann P, Sahl HG (2003). Mutations are involved in emergence of aminoglycoside-induced small colony variants of Staphylococcus aureus.. Int J Med Microbiol.

[11] Clements MO, Watson SP, Poole RK, Foster SJ (1999). CtaA of Staphylococcus aureus is required for starvation survival, recovery, and cytochrome biosynthesis.. J Bacteriol.

[12] Besier S, Ludwig A, Ohlsen K, Brade V, Wichelhaus TA (2007). Molecular analysis of the thymidine-auxotrophic small colony variant phenotype of Staphylococcus aureus.. Int J Med Microbiol.

[13] Chatterjee I, Kriegeskorte A, Fischer A, Deiwick S, Theimann N (2008). In vivo mutations of thymidylate synthase (encoded by thyA) are responsible for thymidine dependency in clinical small-colony variants of Staphylococcus aureus.. J Bacteriol.

[14] Lannergard J, von Eiff C, Sander G, Cordes T, Seggewiss J (2008). Identification of the genetic basis for clinical menadione-auxotrophic small-colony variant isolates of Staphylococcus aureus.. Antimicrob Agents Chemother.

[15] Nagaev I, Bjorkman J, Andersson DI, Hughes D (2001). Biological cost and compensatory evolution in fusidic acid-resistant Staphylococcus aureus.. Mol Microbiol.

[16] Laurberg M, Kristensen O, Martemyanov K, Gudkov AT, Nagaev I (2000). Structure of a mutant EF-G reveals domain III and possibly the fusidic acid binding site.. J Mol Biol.

[17] Chopra I (1976). Mechanisms of resistance to fusidic acid in Staphylococcus aureus.. J Gen Microbiol.

[18] Norström T, Lannergard J, Hughes D (2007). Genetic and phenotypic identification of fusidic acid-resistant mutants with the small-colony-variant phenotype in Staphylococcus aureus.. Antimicrob Agents Chemother.

[19] O'Neill AJ, Chopra I (2006). Molecular basis of fusB-mediated resistance to fusidic acid in Staphylococcus aureus.. Mol Microbiol.

[20] O'Neill AJ, Larsen AR, Skov R, Henriksen AS, Chopra I (2007). Characterization of the epidemic European fusidic acid-resistant impetigo clone of Staphylococcus aureus.. J Clin Microbiol.

[21] O'Neill AJ, McLaws F, Kahlmeter G, Henriksen AS, Chopra I (2007). Genetic basis of resistance to fusidic acid in staphylococci.. Antimicrob Agents Chemother.

[22] Lannergard J, Norstrom T, Hughes D (2009). Genetic determinants of resistance to fusidic acid among clinical bacteremia isolates of Staphylococcus aureus.. Antimicrob Agents Chemother.

[23] O'Brien FG, Price C, Grubb WB, Gustafson JE (2002). Genetic characterization of the fusidic acid and cadmium resistance determinants of Staphylococcus aureus plasmid pUB101.. J Antimicrob Chemother.

[24] Albert TJ, Dailidiene D, Dailide G, Norton JE, Kalia A (2005). Mutation discovery in bacterial genomes: metronidazole resistance in Helicobacter pylori.. Nat Methods.

