# Supplementary material for: Genetic Complexity of Fusidic Acid-Resistant Small Colony Variants (SCV) in Staphylococcus aureus
Source: PLoS One. 2011 Nov 29;6(11):e28366. doi: 10.1371/journal.pone.0028366 (PMC3226684; doi:10.1371/journal.pone.0028366)
Supplement: Table S1 — The following genes including predicted promoter regions (given here with locus tags and product names from the NCTC 8325 genome, GenBank accession number CP000253.1, available at http://www.ncbi.nlm.nih.gov/), were sequenced in all menadione-auxotrophic or hemin-auxotrophic SCV strains. If gene names were not annotated in NCTC 8325 then the names were taken from a closely related S. aureus genome, usually S. aureus COL (GenBank: CP000046.1, available at http://www.ncbi.nlm.nih.gov/). (DOCX) [file pone.0028366.s001.docx]

**Table S1**

| **Menadione biosynthesis-associated genes** | | |
| --- | --- | --- |
| Locus tag | Gene | Product |
| SAOUHSC_00980 | *menA* | 1,4-dihydroxy-2-naphthoate octaprenyltransferase |
| SAOUHSC_00982 | *menF* | Isochorismate synthase family protein) |
| SAOUHSC_00983 | *menD* | 2-succinyl-6-hydroxy-2,4-cyclohexadiene-1-carboxylic acid synthase/2-oxoglutarate decarboxylase |
| SAOUHSC_00984 | *menH* | Alpha/beta fold family hydrolase |
| SAOUHSC_00985 | *menB* | Naphthoate synthase |
| SAOUHSC_01486 | *gerC3* | Heptaprenyl diphosphate syntase component II, putative |
| SAOUHSC_01487 | *menG*, *ubiE* | Ubiquinone/menaquinone biosynthesis methyltransferase |
| SAOUHSC_01488 | *gerC1* | Hypothetical protein |
| SAOUHSC_01916 | *menE* | O-succinylbenzoic acid-coA ligase |
| **Hemin biosynthesis-associated genes** | | |
| SAOUHSC_00509 | *gltX* | Glutamyl tRNA synthetase |
| SAOUHSC_01065 | *ctaA* | Heme A synthetase (putative) |
| SAOUHSC_01771 | *hemL1* | Glutamate-1-semialdehyde aminotransferase |
| SAOUHSC_01772 | *hemB* | Delta-aminolevulinic acid dehydratase |
| SAOUHSC_01773 | *hemD* | Uroporphyrinogen III synthase |
| SAOUHSC_01774 | *hemC* | Porphobilinogen deaminase |
| SAOUHSC_01775 | *hemX* | HemX protein |
| SAOUHSC_01776 | *hemA* | Glutamyl-tRNA reductase |
| SAOUHSC_01960 | *hemG* | Protoporphyrinogen oxidase |
| SAOUHSC_01961 | *hemH* | Ferrochelatase |
| SAOUHSC_01962 | *hemE* | Uroporphyrinogen decarboxylase |
| SAOUHSC_02000 | *hemL2* | Glutamate-1-semialdehyde aminotransferase |
